# Working Conditions in Social Firms and Health Promotion Interventions in Relation to Employees’ Health and Work-Related Outcomes—A Scoping Review

**DOI:** 10.3390/ijerph17113963

**Published:** 2020-06-03

**Authors:** Ann-Christin Kordsmeyer, Julia Christine Lengen, Niklas Kiepe, Volker Harth, Stefanie Mache

**Affiliations:** Institute for Occupational and Maritime Medicine (ZfAM), University Medical Center Hamburg-Eppendorf (UKE), Seewartenstr. 10, 20459 Hamburg, Germany; j.lengen@uke.de (J.C.L.); niklas.kiepe@bgv.hamburg.de (N.K.); harth@uke.de (V.H.); s.mache@uke.de (S.M.)

**Keywords:** employment, health promotion, mental health, occupational health, scoping review, social enterprises

## Abstract

Background: Social firms—a type of social enterprise—offer people with severe disabilities the possibility of employment and integration into the labor market. Since 01 January 2018, social firms in Germany are obligated to provide health promotion interventions for their employees. Therefore, the study aims to provide an overview of the current state of research on working conditions, coping strategies, work- and health-related outcomes, and health promotion interventions in social firms to derive recommendations for action. Methods: The databases PubMed, MEDLINE, PsycINFO, PSYNDEX, CINAHL, and Web of Science were searched. The study selection was based on predefined inclusion and exclusion criteria in the time period between 2000 and 2019. The quality of the studies was critically appraised in a standardized way using the Mixed-Methods Appraisal Tool. Results: A total of 25 studies were included. The current state of research indicated that employees with disabilities were provided with several environmental resources like social support, flexibility, structured work tasks or options for training. A mix of environmental and personal resources impacted several work- and health-related outcomes like well-being, job satisfaction, productivity, work engagement, the motivation to work, or job tenure. Conclusions: There is a need for further (longitudinal) research concerning the work and health situation of employees working in social firms and the development of health promotion interventions.

## 1. Introduction

For people with disabilities, employment provides plenty of benefits, but it is also associated with some challenges. Maintaining employment in the competitive labor market represents an important factor in the recovery process for people with disabilities [1,2]. Basic routines in combination with appropriate work accommodations enable employees to develop work-related self-esteem and social skills and may decrease hospitalizations [3]. Moreover, individual control and empowerment, as well as higher levels of autonomy and self-determination, can be promoted [3]. Therefore, a social and work-related identity—how a person defines themselves at work and in the environment—can be developed, which encourages a feeling of being a member of the community [3]. Barriers to employment result from social stigma and discrimination, a lack of options to work or interfering symptoms, e.g., of severe mental health conditions [4,5]. Those circumstances are also reflected in rates of employment of people with disabilities which are much lower compared to the general population (e.g., 46.9% compared to 75.2% in 2017 in Germany [6]), although the majority is able and would like to work [4,7].

People with disabilities have several options to gain employment, including, e.g., sheltered workshops and supported or transitional employment [8]. However, in this study, the focus is on social firms—a type of social enterprise (known as a business following social objectives, achieving their income from sales instead of government subsidies and reinvesting their profit in the business or community-related activities without aiming at individual gain) [9]. Depending on company objectives, management techniques, and the country, social firms are also called “affirmative businesses, adapted enterprises, cooperatives, collectives, [or] consumer/survivor-run businesses” ([1], p. 39). Developed in the 1970s in Italy, the employment model has spread throughout Europe. Moreover, affirmative businesses gained prominence in East Asia and North America as an independent development [8].

Corbière et al. (2019a) summarized that all of the mentioned employment models follow the same key principles regardless of the country: First and foremost, they are defined as non-profit businesses generating meaningful employment for disadvantaged persons in the open employment market (social objective). A significant number of people with disabilities or otherwise disadvantaged persons (at least 25%) is employed and provided with participative decision-making structures, the promotion of autonomy, and self-empowerment while working together, as well as increased levels of social support promoting a sense of community. They aim at financial viability, preferably apart from subsidies or external sources [1]. Referring to the German context, social firms must employ at least 30% and can employ up to a maximum of 50% people with severe disabilities. People with different types of disabilities are employed, as well as those who are in transition between a sheltered workshop and a company in the open employment market. In addition, long-term unemployed people with severe disabilities as well as people who have completed special education find work here (Section 215, paragraph 2 of the German Social Insurance Code IX).

With the legislative changes of January 1, 2018 in Germany, social firms are obliged to take measures for workplace health promotion. This represents a significant difference to other companies in the open employment market. However, only little research has been conducted evaluating workplace health promotion interventions within vocational rehabilitation. Research activities on this topic are elementary because people with disabilities experience poorer health than the general population: People with disabilities assess their physical health status and psychological well-being substantially lower than people without disabilities [10,11]. Furthermore, less physical activity and, in some cases, overweight and alcohol and tobacco consumption are reported [10,12]. The workplace provides an adequate setting to integrate health promotion interventions into the everyday lives of employees (see Ottawa Charta [13]), since people with disabilities are often hard to motivate [14]. Starting points for health promotion interventions are focused either on behavior-related approaches which aim at encouraging healthier lifestyles (e.g., through healthier eating habits, physical activity, and enhanced stress management strategies) or on structural approaches (e.g., improved working conditions or organizational interventions) [15].

Results suggest that serval workplace health promotion interventions have been identified as impacting employees’ health in a positive way (e.g., cognitive-behavioral interventions or mindfulness trainings were evaluated to be effective in decreasing depression, anxiety, or burnout and increasing employees’ well-being) [16]. However, present interventions were often not adapted to the specific requirements of employees with disabilities [14] or settings like social firms. Therefore, the scoping review aims to analyze the current state of research on working conditions, coping strategies, work-related outcomes, and health promotion interventions in social firms to derive recommendations for action. Results will be mapped and visualized for policy makers or responsible persons in social firms. Moreover, research gaps can be identified.

For exploring the current state of research, the occupational psychological stress model of Bamberg et al. (2003, 2006) is considered as a theoretical framework for the scoping review [17,18]. Elements of the stress and strain concept of Rohmert and Rutenfranz (1975) are considered and supplemented by the transactional stress model of Lazarus (1974) [19,20]. Various starting points for further analyses and interventions can be derived in a systematic way as the model distinguishes between environmental demands and resources, as well as personal risk factors and resources, and includes processes of appraisal (primary and secondary), coping strategies, and subsequent outcomes. Repercussions between these characteristics and processes were stated as they impact or reinforce one another.

## 2. Materials and Methods

The framework underlying the scoping review referred to Arksey and O’Malley (2005) to examine the extent of scientific research, identify gaps in research, and derive recommendations for future research [21].

### 2.1. Identifying the Research Questions

According to the theoretical framework of Bamberg et al. (2003, 2006) the scoping review took the following research questions into account to analyze working conditions, coping strategies, employees’ health- and work-related outcomes, and health promotion interventions in social firms:

1. Which environmental and personal resources are available for employees in social firms?
2. What kind of environmental job demands and personal risk factors are identified for employees in social firms?
3. Which coping strategies do the employees in social firms use when dealing with job demands?
4. What work- and health-related outcomes for employees in social firms are reported?
5. Which workplace health promotion interventions are implemented in social firms?

### 2.2. Identifying Relevant Studies

To identify relevant studies, six electronic medical and psychological databases were selected: PubMed, MEDLINE, PSYNDEX, PsycINFO, Web of Science, and CINAHL. The databases were searched between January 2000 and December 2019. Table S1 (Supplementary Materials) indicates the search strategy, which was developed for PubMed and afterwards adapted for the other databases. Two key topics were combined: (1) different names for the considered vocational setting (see [1]) and (2) environmental and personal resources, job demands, and personal risk factors; coping strategies; work- and health-related outcomes; and health promotion interventions. Afterwards, reference lists of eligible studies were searched for further relevant studies.

### 2.3. Study Selection

The study selection was based on predefined inclusion and exclusion criteria. Studies were included when a significant number of people with disabilities or otherwise disadvantaged persons were employed in a social firm or social enterprise paying a fair remuneration (market rate wage or salary appropriate to the sector). Settings that were explicitly identified as emerging social firms were additionally included. Furthermore, an examination of the main outcomes regarding job demands, resources, work- or health-related outcomes, coping strategies, or health promotion interventions should be provided. Studies dealing with supported employment programs or sheltered workshops were excluded. Overall, only studies in English or German language were included.

Different approaches were considered for the review including quantitative, qualitative, and mixed-method studies. Title and Abstract Screening was conducted by one reviewer. Full texts were screened for eligibility by two reviewers independently (A.C.K. and N.K.). The inter-rater reliability was calculated using Cohen’s kappa. If no decision could be made, another reviewer was considered.

### 2.4. Charting the Data

Based on the approach of Arksey and O’Malley (2005), information of eligible studies was charted considering the author, title, year of publication, country, study design, methodology, study population, setting, aims of the study, outcomes, and important results (Supplementary Materials, Table S2). Data were obtained and compared by two reviewers. Main results of the scoping review were analyzed using a deductive approach according to the research questions (main categories: job demands, personal risk factors, environmental and personal resources, coping strategies, work- and health-related outcomes, and health promotion interventions) and categorized using MAXQDA (version 12). Moreover, significant statistical estimates (* *p* < 0.05; ** *p* < 0.01) of cross-sectional and longitudinal studies conducting different multiple regression analysis techniques were extracted and visualized.

### 2.5. Collecting, Summarizing, and Reporting the Results

Based on the additional recommendation of Levac et al. (2010), three steps were taken into account for summarizing and reporting the results: Initially, an analysis including descriptive and numerical data was carried out, followed by qualitative content analysis techniques to illustrate the main results of the scoping review in a narrative way. Afterwards, the results were reported and referred to the main objectives and research questions of the scoping review. Finally, a discussion was provided and implications for further research, practice, and policy makers were drawn [22]. Results were presented according to the PRISMA extension for scoping reviews (PRISMA-ScR) by Tricco et al. (2018) providing essential reporting items [23].

### 2.6. Quality Assessment

Currently, there were no consistent recommendations for quality assessment in scoping reviews available [22]. Based on the suggestions of Brien et al. (2010) a quality assessment was conducted evaluating the methodological depth of the included studies to facilitate the interpretation of the results [24]. Hence, two reviewers examined the methodological quality of the included studies in a systematic way using the Mixed-Methods Appraisal Tool (MMAT) provided by Hong et al. (2018) [25].

The MMAT is an appraisal tool which can be used to examine the methodological quality of different study types (qualitative research, randomized controlled trials, non-randomized studies, quantitative descriptive studies, and mixed-methods studies). To assess the quality of mixed-method studies, the quality of qualitative and quantitative components should be taken into account [25].

## 3. Results

**Figure 1:**
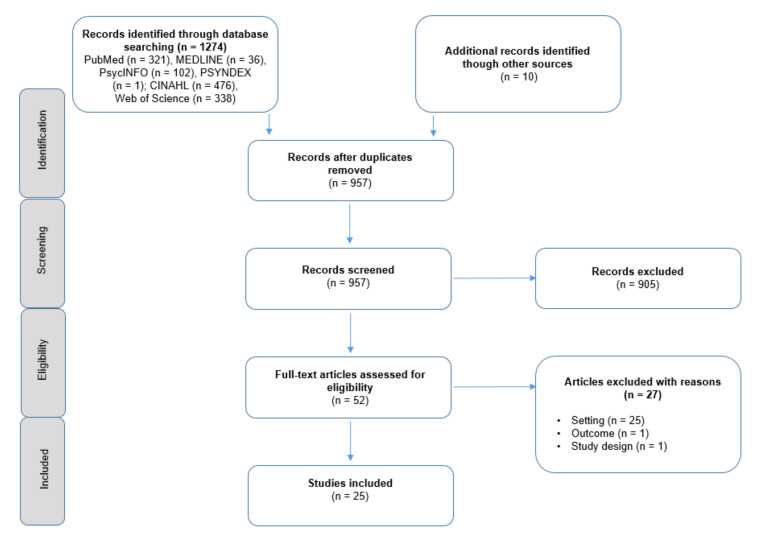
PRISMA Flow Diagram for the selection process.

In total, 1274 records were found through database screening. After the screening of titles and abstracts, 52 records were assessed for eligibility. Articles were excluded with regard to the setting, e.g., when no market rate wages or salaries appropriate to the sector were payed (*n* = 27). Moreover, one study was excluded due to an unsuitable outcome and a lacking connection to the research questions. Other reasons for exclusion referred to the study design (e.g., reviews, *n* = 1). The moderate Cohens-Kappa of 0.73 shows a substantial agreement between the two reviewers (A.C.K. und N.K.) according to Landis and Koch (1977) [26]. Figure 1 illustrates the process of study selection. 

### 3.1. Study Characteristics

While the majority of studies predominantly analyzed the work and health situation of target staff with mental disorders working in social firms or social enterprises [1,27,28,29,30,31,32,33,34,35,36,37,38,39,40,41,42,43,44,45,46,47], three studies reported additional employment opportunities for people with learning, sensory, intellectual, or developmental disabilities; or those suffering from long-term unemployment [48,49,50]. Fourteen studies reported evidence gained from the target group itself [28,30,31,32,33,34,35,36,37,38,39,40,41,46], nine studies considered a mixed sample (e.g., of employees, managers, or support staff/carers [1,29,42,43,44,45,47,48,50]), and two studies consisted of a sample of non-disabled participants as key informants (executive directors or managers) [27,49]. Socio-demographic characteristics of the included studies indicated that most participants were male (51–93.75%), except in three studies [30,31,33] (not specified in nine studies [27,29,41,42,43,44,45,49,50]). The age of participants ranged between 35.5 (median [34]) and 48 years (mean [30]) [1,28,30,31,32,33,34,35,36,37,38,39,40,41,46,47]. Most of the studies (*n* = 15) reported that employees were working primarily on a part-time basis (less than 35 h) [27,29,31,32,33,35,36,37,40,41,43,45,47,48,49]. The job tenure ranged between 15 months (median, [34]) and 7.3 years (mean, [30,37]), whereby seven studies reported an average length of employment of over 5 years [1,28,30,31,35,37,39]. Further characteristics of the included studies are displayed in Table 1.

### 3.2. Quality Assessment

The results of the quality assessment using the MMAT [25] are illustrated in Table S3 (Supplementary Materials). Most of the qualitative studies met all the criteria of the MMAT (*n* = 8). In contrast, quantitative descriptive and mixed-method studies were of lower quality according to the MMAT. Eight studies with a quantitative descriptive approach, as well as the three mixed-method studies, met two of the five quality criteria. Examining the inter-rater reliability for the quality assessment a Cohen’s Kappa of 0.70 indicated a substantial agreement between the two reviewers [26].

### 3.3. Environmental Resources

Following the theoretical framework, social support, flexible working arrangements, structured work tasks, training opportunities, payment, job security, and participation were identified as fundamental environmental resources in the current body of evidence.

#### 3.3.1. Social Support

A range of supportive social interactions at work were described most often in the current state of research, including interactions with supervisors, the natural support of co-workers, customer or community interactions, and those related to organizational or agency partnerships [1,27,28,29,30,32,33,34,35,36,37,38,39,40,41,42,43,44,45,47,48,49,50]. Furthermore, the relevance of feedback and appreciation was stated [1,37] and exemplified in two ways, either formally (e.g., when conducting worksite inspections) or informally (e.g., during conversations or customer interactions) [40], which supported employees’ performance and satisfaction [40,41]. Moreover, supporting new co-workers (e.g., by means of a formal mentorship [45]) allows employees to independently reflect on their own (working) progress and increasing skills [34] with a positive impact on job satisfaction [40,41]. Overall, the development of social relationships at work could also be enhanced by different kinds of social events [30].

Additionally, the social support from supervisors was pointed out as a key resource promoting acceptance and inclusion, applying fair management techniques [30,34,40,41]. Supervisors could shape an environment of learning from previous mistakes, paying attention to how employees are doing at work, and providing practical assistance [27,30,34,49]. Being able to do so, Corbière et al. (2019a) highlighted that the participating supervisors received training about mental health conditions in Québec (in adapted enterprises, which have to employ at least 60% of staff with a disability) and in Ontario (in consumer/survivor-run businesses, which were developed and run by people with a mental health condition) [1].

Moreover, receiving support from different stakeholders like peers, friends, family, a mentor, or employment specialist was considered as useful but less available in two types of Canadian social firms. The provision of a mentor was the most frequent accessible source of support [1]. Results were in line with Villotti et al. (2017), who also reported moderate access of the support from different stakeholders referring to one type of occupational setting (cleaning services) [37].

Additional support with challenges of the daily life were discussed in the literature [27,45,49] including assistance with housing, transportation, or welfare benefits. The authors described that it might not be a conventional accommodation, but it had advantages for the individual, resulting in positive organizational outcomes like a more permanent workforce [27,45,49].

To sum up, being able to work in a supportive atmosphere within a benevolent social group contributed to social inclusion and a sense of belonging and promoted a shared and individual work identity [29,30,32,34,42,50].

#### 3.3.2. Flexible Work Arrangements

The second most stated environmental resource referred to flexible work arrangements, mainly in the context of scheduling and work tasks [1,27,29,30,32,33,34,37,40,41,44,45,47,49]. According to Corbière et al. (2019a) the schedule flexibility especially for medical reasons was high (62.9–78.3% depending on company type) and identified as being useful [1]. Comparing work accommodations in three countries (Italy, Canada, and Australia), Villotti et al. (2017) reported schedule flexibility as the most frequently implemented resource with a positive impact on job tenure [37].

Williams et al. (2010, 2012) added that regular part-time hours and working on rotating days combined with some flexibility in the schedule were described as having a positive effect on performance, well-being, and job satisfaction in the workforce of a single social firm [40,41]. Moreover, target group employees were able to discuss leaves of absence, desired working hours, or shifts depending on several factors like the mental and physical health situation, health care needs, appointments or limits resulting from disability benefits, family obligations, or other activities like social or sports clubs [27,29,32,40,41,44,45,47,49]. The variations regarding provided flexibility was large: Some companies were able to offer complete flexibility and fulfil the working time wishes of their employees, others determined a minimum of working hours according to their business requirements [27,49].

Furthermore, flexibility in the context of work tasks was emphasized. It included regular tasks and locations and the opportunity to negotiate changes (“we adapt the work to the worker, not the worker to the work” ([49], p. 569)). The target group tended to develop routines, felt comfortable in familiar workplaces, worked at their own pace, were able to take unscheduled breaks, and were not put under pressure [29,34,40,49]. A prerequisite for this kind of flexibility is the willingness to divide or reallocate tasks among different employees as well as to adapt staffing related to work tasks or settings [27,47,49].

#### 3.3.3. Structured Work Tasks

A structured activity was described as having something to do and somewhere to go pursuing a noticeable objective [34,45]. In social firms, it should be ensured that work is perceived as meaningful, which was described as being fundamental to the development of a work identity and underlined by the demand for the produced goods and services or by payment [29,34,42,45,48]. When starting to work in a social firm, the process of an on-the-job negotiation was described identifying appropriate tasks and duties which fit to the employee’s skills, capacities, and interests [27,33,45]. When finding suitable activities, it was highlighted that supervisors may need to adapt expectations regarding the work pace of the employee and to not put too much pressure or high demands on the employee [27,30,49].

Over time, skills development was mentioned as a crucial factor for future career steps providing suitable challenges for employees, including an ongoing monitoring and evaluation of individual progress [32,34,48]. Moreover, participants described themselves as experienced when they were involved in regular duties or when following responsibilities, like supporting new employees, locking something up, or other checking activities [40]. The satisfaction of employees with their tasks was promoted by training courses and additional responsibilities like working with little supervision [41]. Other approaches to reward work experiences or performance were stated by Lysaght et al. (2018) referring to foreman positions overseeing attendance, holiday schedules, or other routines.

Overall, a consistent and structured job activity, with the perception of a “regular” business regarding its structures, processes, standards, or customer service, was described as a contributing factor to self-esteem and competence, community participation, and the worker’s identity [32,34,40,41,42,44,45] with an additional positive impact of stigma reduction of mental illness [43].

#### 3.3.4. Training

In the current state of research, the relevance of training possibilities was described in promoting a sense of expertise [1,30,37,40,48]. Corbière et al. (2019a) highlighted differences in the provision of training for employees in two types of Canadian social firms, like the gradual introduction of tasks, access to educational resources, the adjustment to the learning pace, or the training of self-management and communication skills (84.9% in survivor-run businesses and 68.7% in adapted enterprises). Analysis revealed that the majority of employees reported that it would be a useful work accommodation [1]. Results were in line with Villotti et al. (2017) who reported a moderate implementation of work accommodations related to training in Australian, Canadian, and Italian social enterprises. Overall, training possibilities were associated with an increased job tenure [37].

Moreover, job coaches were often present in adapted enterprises (93.1%), but less available in consumer-run businesses (33.8%), although this work accommodation was perceived as useful by both types of social firms [1]. To promote learning and personal development of employees, cooperation with training and education agencies was needed according to Secker et al. (2003). A model which was highlighted included a designated staff member who was responsible for employee development in social firms [48].

#### 3.3.5. Payment and Job Security

Benefits of the job like payment or job security were rated as strongly supportive for the workers job satisfaction, well-being, social confidence, and quality of work life [30,40,41,45,47,50]. Pay and conditions in social firms were perceived as a bonus, especially in comparison to other jobs available in the open employment market [40,41]. With the aid of regular wages complementing disability benefits, employees were more financially independent (e.g., being able to save money or enjoy leisure activities) which increased social participation [29,40,41,49,50]. Additional advantages contributing to the employee’s satisfaction included, e.g., paid annual leave, public holidays, or pension [41].

Moreover, employees benefited from an increased job security with additional unpaid sick leave in Australian results [40,41]. The factor security was also mentioned as a central work accommodation for both short-term and long-term employee absences due to health circumstances. The supportive environment of social firms was especially described as supervisors being understanding about short-notice cancellations of shifts in case an employee does not feel well [27,29,32,45,49]. It was also stated that jobs were held for many employees during long-term absences for (physical or mental) health-related reasons [27,45,49].

Although participants viewed their jobs as secure, advancement possibilities were described as limited [40,41,47]. The majority of people work part time and earn above-minimum wages with low prospects of higher wages [27]. Although this type of employment seems to decrease a financial dependency, it was not fully resolved [42,43].

#### 3.3.6. Participation

Several authors underlined the relevance of participation, e.g., as increasing someone’s work identity and social skills [29,33,42,48,50]. Six of 29 participants in a study conducted by Secker et al. (2003) reported that social firms were initiated considering the potential worker’s situation and related needs. When operating such employment models, managers highlighted regular meetings, workshops, committees, or comparable formats as opportunities to participate in decision-making processes [48]. The use of worker advisory committees or close meeting and communication structures were also found in other studies, as well as family engaging structures [27,42,50]. Paluch et al. (2012) reported that workers did not always feel like being involved in decision-making processes of the business, in which case regular debriefing sessions were suggested [33]. Other participants reported that employees were able to influence decision-making processes more informally. However, it was not clear to what extent these formal and informal approaches encouraged employee participation, since workers of social firms were not included in the study [48].

### 3.4. Personal Resources

#### 3.4.1. Occupational Self-Efficacy

In the included studies, the personal resource of occupational self-efficacy was frequently mentioned [30,35,36,38,39]. Gaining insight into vocational outcomes, Villotti et al. (2012) reported that employees who express work-related confidence in dealing with the demands in social enterprises were more likely to be satisfied. Moreover, occupational self-efficacy was also related to all three dimensions of work engagement (vigor critical ratio (CR) 4.31, dedication CR 3.77, and absorption CR 4.53) [38]. A positive link to motivation to work was also found in path analysis [35].

As part of a serial mediation analysis, job tenure self-efficacy (described as the individual ability to deal with work-related demands with a focus on keeping employment) was also identified as a mediator in the relationship between social support and productivity (specific indirect effect 2.18 confidence interval (CI): 0.73–4.71) [39].

#### 3.4.2. Work-Related Self-Esteem

In a longitudinal study of Corbière et al. (2019b), a positive influence of work-related self-esteem on productivity was reported at a baseline survey. However, after six months, follow-up results indicated work-related self-esteem neither as a predicting factor of work productivity [28] nor as a predictor of job tenure [31].

### 3.5. Job Demands

#### 3.5.1. Job Tasks

Overall, only a few job demands were presented in terms of job tasks. Lanctôt et al. (2012b) described a balance in the selection of favorable and unfavorable job tasks for the employee with disabilities. Depending on individual preferences, some employees favored recurring job tasks, while others preferred more challenging tasks [30]. Williams et al. (2012) added that participants assessed physical and cognitive job demands as achievable with adequate challenges.

#### 3.5.2. Work Environment

Job demands due to the work environment referred to, e.g., noise or physical constraints [30]. More specifically, Williams et al. (2012) reported environmental demands such as working in hot offices without air conditioning in a social firm providing cleaning services [41]. Two studies reported that work accommodations due to the work environment were less available for employees in social firms [1,37]. Comparing the results in two types of Canadian social firms, it turned out that about 60% were able to modify the work environment (e.g., changing levels of noise). However, the majority of employees reported that changes in the work environment would be a useful work accommodation [1]. Qualitative analysis of a single social firm revealed that equipment, materials, or being able to have a seat were mentioned as enhancing the quality of work life [30].

#### 3.5.3. Social Relations

In the context of job demands referring to social relations, feelings of becoming a burden to the team due to not completing tasks in time were mentioned as burdensome [41]. Moreover, harassment was highlighted as having an impact on the perceived quality of work life in qualitative analysis [30].

Additional results were reported indicating job demands which may arise when employees’ comfort with social interactions was not considered enough [27,44,49]. On the one hand, this may refer to the work in customer service positions, on the other hand, it was relevant within social interactions with co-workers, e.g., if someone had a bad day and needed space for him- or herself. In general, the majority of managers were aware of those demands and maintained, e.g., additional staff [27,44,49].

Other tensions may arise when competences of employees were medicalized in a way that employees were perceived as patients rather than employees (e.g., when customers act in an overly careful way) [42,43] or persistent stereotypes during customer encounters [44].

#### 3.5.4. Organization of Work

Job demands due to the organization of work in social firms referred mainly to working hours and a high workload. A central topic in the context of working hours was the dissatisfaction with limited working hours (e.g., only up to 15 h) for holding disability benefits [27,29,41]. Moreover, irregular changes of working hours or late afternoon shifts were reported as demanding [41]. From an organizational perspective, interpersonal conflicts were reported when distributing (limited) working hours among employees, e.g., when assigning extra shifts [49].

Other job demands in the context of work organization resulted from a heavy workload or time pressure [28,35,36,41]. High levels of organizational constraints were reported as having a negative impact on work productivity [28], job satisfaction (odds ratio, OR, 0.944 CI 0.905–0.985) [36], and the motivation to hold the job [35]. Focusing on profit maximization only may influence organizational constraints and therefore organizational outcomes. Especially supervisors faced a two-folded challenge in managing both economic and social processes [27,33,43,45,49].

### 3.6. Personal Risk Factors

#### 3.6.1. Severity of Symptoms

Severity of symptoms was researched by several authors who investigated the effects of psychiatric symptoms in the context of supportive work in social firms. In this construct, different symptoms like somatization, depression, or anxiety were assessed for calculating a global severity index. In a longitudinal study, Corbière et al. (2019b) found a negative impact of the severity of symptoms on work productivity at baseline, but not after six months follow-up. However, results were not confirmed by the study of Villotti et al. (2018b), assuming a significant negative relationship between severity of symptoms and work productivity after 12 months (modified model). Moreover, severity of symptoms was also negatively related to the work engagement dimension vigor (comprising of energy and endurance at work, CR–2.02) [38]. However, job satisfaction [36], the motivation to keep employment [35], and job tenure [31] were not significantly predicted by an employee’s severity of symptoms.

#### 3.6.2. Self-Stigma

Villotti et al. (2018a) identified self-stigma as a mediator in the relationship between social support and perceived work productivity with a specific indirect effect (1.87 CI: 0.55–3.84). Therefore, the authors highlighted the relevance of providing a supportive work environment for minimizing self-stigma and increasing the employee’s confidence in dealing with work-related demands [39].

Krupa et al. (2019) added that the extent to which a business is perceived as contributing to the community affects also workers’ self-stigma, when identifying themselves as valued. Despite comprehensive support, fragile levels in self-evaluations were highlighted assuming ongoing levels of self-stigma (“I’m good for nothing; I’m not intelligent; I’m not capable; I can’t, do anything.”) ([43], p. 487).

### 3.7. Coping

#### 3.7.1. Emotion-Oriented Coping

Gaining insight into emotion-oriented coping strategies of employees in social firms, it turned out that the studies focused on coping with mental illness at work in the first place [29,33,34]. Svanberg et al. (2010) emphasized in a sample including predominantly male participants that being active, getting up in the morning for work, and developing individual goals contributed to the process of recovery. The target group reported various coping strategies like separating oneself from their mental illness in order to control symptoms [34]. Krupa et al. (2003) highlighted approaches for self-management in dealing with cognitive deficits or social discomfort. Another topic without consensus in social firms was the information policy of the employees regarding the disclosure of a (mental) health condition. Paluch et al. (2012) stated that “stepping around peoples’ mental illness could obscure the issue, yet ‘knowing’ about it could lead to judgments and assumptions about work performance” ([33], p. 68). On the contrary, communicating relevant information was described as necessary for gaining a sensitive response from colleagues and supervisors, meeting the employees’ needs, and maintaining a supportive environment [33]. In general, openness and acceptance of mental health conditions were highlighted ensuring a comprehensive discourse of an employee’s personal needs [30,32,33,34,44].

#### 3.7.2. Problem-Oriented Coping

Focusing on problem-oriented coping strategies, the relevance of social support was already pointed out when dealing with work-related demands. Moreover, two studies highlighted the use of medication as a strategy maintaining control over symptoms of mental illness [29,34]. Thereby, employees were able to stay active and enhanced the capability to work. In this context, results from two emerging social firms in Scotland [34] underlined that mental health services were commonly perceived as beneficial. Some participants reported that they needed both the support of social firms and mental health services, being able to speak to professionals. Others reported that mental health services only provided medication or that “social firms were able to pick up where mental health services left off” ([34], p. 488). However, a close relation to the mental health system, may influence the worker’s identity or create barriers to community participation increasing the impression of a business as a “service” in place of a regular business [42,43].

**Figure 2:**
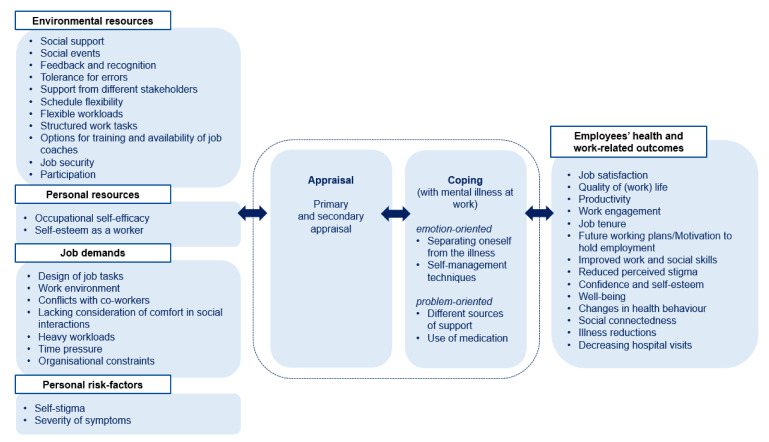
Overview of the work and health situation of employees in social firms (own figure based on [17,18], adapted version).

To sum up, results referring to environmental and personal resources, job demands, personal risk factors, applied coping strategies, and the following work- and health-related outcomes were integrated into the structure of the occupational psychological stress model of Bamberg et al. (2003, 2006) and are visualized in Figure 2.

### 3.8. Work and Health-Related Outcomes

#### 3.8.1. Work-Related Outcomes

Based on the current state of research, several work-related outcomes were examined in the context of social firms. First of all, a logistic regression was used to gain insight into job satisfaction. Employees scoring higher on occupational self-efficacy (OR 1.586 CI 1.198–2.101), who were equipped with workplace accommodations (OR 1.148 CI 1.012–1.303) and social support (OR 1.533 CI 1.031–2.279), were more likely to express job satisfaction. No significant relationship was found for external support, e.g., from the family, or severity of symptoms [36]. Mean scores on overall job satisfaction were also high in a study conducted by Milton et al. (2015) (5.87, standard deviation (SD) 0.89, maximum score of 7).

Furthermore, results from Lanctôt et al. (2012a) demonstrated the impact of quality of work life on job tenure; 89% of the participants maintained their job during the follow-up period (six months). Results indicated that employees with a higher quality of work life had a decreased risk for job loss (hazard ratio (HR) 0.005, CI 0.00–0.77, *p* = 0.039). Severity of symptoms, self-esteem as a worker, job satisfaction, age, and general quality of life were not verified as other predictor variables [31]. However, the study referred to a relatively small sample size (n = 67). Job tenure was also examined in the Australian, Canadian, and Italian context. Villotti et al. (2017) illustrated that particularly training possibilities and schedule flexibility were associated with an increased job tenure in a multiple regression analysis [37]. Older age (0.237, *p* = 0.034) and country of origin (Australia vs. others; −0.329, *p* = 0.010) were the only control variables considered to be significant [37]. To shed more light on the motivation to work, results indicated a positive influence of occupational self-efficacy and social support and a negative one of organizational constraints. Further analysis after 12 months follow-up revealed enhanced social and work skills which in turn lead to a higher perception of productivity and lower levels of perceived stigma (path analysis, modified model) [35].

Furthermore, work engagement was analyzed in a longitudinal study considering three dimensions: vigor was described as having high levels of work-related energy and endurance; dedication included a strong sense of belonging, enthusiasm, inspiration, and pride; and absorption was related to concentration at work [38]. Employees who received social support from co-workers and supervisors were dedicated (CR 2.81) and absorbed in work-related goals (CR 2.87). Likewise, the work engagement dimension vigor had also a positive impact on future work plans after 12 months in path analysis as a main goal for the target group (CR 2.83) [38].

In the current state of research, different longitudinal analysis examined productivity in social firms [28,35,39]. Based on a serial mediation model, Villotti et al. (2018a) found that social support resulted in increased perceptions of productivity fully mediated through lower perceived stigma and higher levels of job tenure self-efficacy (1.01 CI 0.42–2.28) after six months follow-up. Therefore, the authors concluded that discriminating or non-supportive work environments increased the level of self-stigma, which in turn reduced individual confidence in dealing with work-related problems influencing productivity [39]. The relevance of supportive workplaces in the context of work productivity was also underlined in path analysis by Corbière et al. (2019b). The authors analyzed a theoretical model for work productivity, considering health, individual, and organizational aspects and controlling for province, gender, age, diagnosis, and job tenure (not significant in the model tested). Compared to the lacking influence of supervisor support at baseline, it was the only factor which predicted work productivity after six months (adjusted model) [28]. Neither of the described surveys used randomly selected participants, but rather referred to convenience sampling [28,31,35,36,37,38,39].

**Figure 3:**
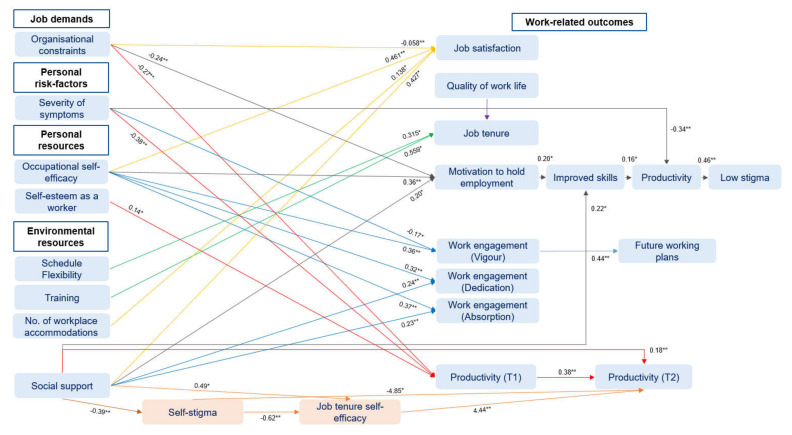
Overview of quantitative analysis concerning working conditions in social firms. * *p* < 0.05; ** *p* < 0.01 (orange, *n* = 170 [39], blue, *n* = 121 [38], red, *n* = 222 [28], yellow, *n* = 248 [36], green, *n* = 90 [37], purple, *n* = 67 [31], grey, *n* = 139 [35], own figure).

To sum up, Figure 3 includes an overview of significant statistical estimates of the included studies using multiple regression analysis techniques (* *p* < 0.05; ** *p* < 0.01). Predictor and outcome variables (blue) were presented as well as mediation variables (orange).

#### 3.8.2. Health-Related Outcomes

As already presented, achievable job tasks combined with a regular structure and (flexible) work schedules, supportive workplace interactions, and the provision of workplace accommodations were described as contributing to satisfaction, community participation, perceived self-confidence, well-being, and quality of work life, providing a basis for recovery from mental illnesses [29,30,32,34,40,41,47,50].

First results of a three-stage mixed-methods longitudinal case study by Elmes et al. (2019) added evidence on health-related impacts. Data revealed that most of the participants reported improved health compared to a year ago, changes in health behavior (e.g., weight loss or quitted smoking), increased social connectedness and well-being by means of advanced living conditions and financial circumstances, and amplified levels of confidence and self-esteem based on positive work experiences. Nevertheless, about half of the target staff stated high or very high levels of distress [47].

In line with a study from the UK [32], a reduction in hospital visits was presented since being employed [47]. In the same way, Milton et al. (2015) demonstrated significant reductions in employees self-reporting various mental health conditions, self-harm, and medication intake. Moreover, results indicated a reduced self-reported contact to counsellors or psychotherapists, psychiatrists, or crisis teams [32]. Results were partly confirmed in Canadian results, comparing social enterprise employees with a non-employed group with severe mental disorders. During the last six months, employees of the social enterprise group were significantly less likely to be hospitalized for a psychiatric reason (OR 0.14 CI 0.037–0.53) or obtaining community mental health support visits (OR 0.41 CI 0.20–0.85). No significant results were observed considering primary care, psychiatrist visits, and the use of medication, as well as for emergency room visits after adjusting [46].

While past experiences of unemployment were characterized by feeling bored, apathetic, isolated, or inactive in combination with excessive sleep, current working conditions were associated with a reduction of depression and severity of symptoms in qualitative results [29,45]. Work was compared with some kind of therapy dealing with mental health conditions supported by several work accommodations [29,44].

### 3.9. Health Promotion Interventions

Within the scope of the review, no studies dealing with workplace health promotion interventions in the setting of social firms or social enterprises were identified.

## 4. Discussion

In this scoping review, the current state of scientific research regarding working conditions (job demands and resources), coping strategies, work- and health-related outcomes, as well as workplace health promotion interventions in social firms was examined. Results were mapped, visualized, and integrated in the theoretical framework of Bamberg et al. (2003, 2006). The majority of research was focused on environmental resources like flexible working arrangements or social support and work-related outcomes like productivity or job satisfaction. Only little research investigated job demands, coping strategies, and health-related outcomes.

For employees with mental health conditions, being able to hold employment represents an important objective. The study results indicated a maximum job tenure of 7.3 years on average [30,37]. Other studies reported that most of the employees with mental illnesses had been employed for over 2 years [9]. Therefore, in terms of job tenure, social firms appear to be the more stable type of employment [1,9,31] compared to supported employment programs with a main job tenure of only a few months as shown in two recent meta-analyses [51,52]. These tendencies are supported by a study exploring working plan patterns of employees with mental health conditions in Italy, deriving that 57.7% showed a strong intention to hold their job in social enterprises [53].

### 4.1. Working Conditions in Social Firms

Results indicated that working in a social firm was characterized by the provision of several environmental resources depending on the occupational setting, the country, and management-related factors. The most popular environmental resources referred to social support [1,27,28,29,30,32,33,34,35,36,37,38,39,40,41,42,43,44,45,47,48,49,50], flexible work arrangements [1,27,29,30,32,33,34,37,40,41,44,45,47,49], structured work activities [27,29,30,32,33,34,40,41,42,43,44,45,48,49,50], payment and job security [27,29,30,32,40,41,45,47,49,50], or the provision of trainings [1,30,37,40,48]. Similarly to the presented results on social support, a study by Chan (2015) revealed that social support should consider task-related demands, interpersonal conflicts, non-vocational barriers for cultivating employees’ self-confidence (e.g., by means of additional coaching or forwarding to other services) [54]. Further analysis affirmed that practical social support increased former low levels of optimism and self-esteem of workers, especially for those reviewing the support as beneficial. Thus, the provided social support may enable the development of personal resources with the potential of increasing optimism and self-esteem in promoting resilience [55].

To be able to work with employees with a mental health condition, an understanding of how to accommodate is necessary because specific needs appear less noticeable or complex [37]. Since high levels of supervisor support were also described as affecting work productivity in the long run (but not at baseline), an accumulation of supervisor support after a while was assumed, when they became familiar with each other and know individual needs for support [28]. One example to support supervisors in social firms was the provision of training, e.g., regarding mental health conditions [1,30]. As can be seen from results of the general population, a positive health effect of specific management styles was assumed in previous research [56]. Therefore, this topic should be considered in future analyses in social firms either referring to outcomes like job tenure [37] or health-related outcomes.

In the context of flexible work arrangements, there were differences between social firms concerning the levels of flexibility. Some social firms were able to maintain complete flexibility regarding weekly working hours, while other businesses scheduled a certain number of hours per week [1,27,49]. This may be influenced by business-related factors on the one hand and health-related factors of an employee on the other hand [27]. The provision of trainings (e.g., gradual introduction of tasks, access to educational resources, or the training of self-management and communication skills) also played a prominent role in the context of environmental resources [1]. More interventional research is needed evaluating different concepts for training. Thus, suitable offers for employee development considering the diverse needs for workers with and without disabilities can be provided. Currently, there are no guidelines or regulations leading employers to the implementation of environmental resources supporting employees with a disability [37]. Overall, a participative work environment should be implemented in the social firm’s culture and management styles increasing employee’s influence in decision-making. Since supervisors and managers are not always prepared to provide participatory structures, or business- and productivity-related demands may hinder such processes, guidelines could contribute to a framework for responsible persons and other stakeholders involved [33].

According to the second research question, several environmental job demands were identified in varying occupational settings. The settings were traditionally described to be limited to the “4F jobs” (food (gastronomy), filth (cleaning), filling (packaging), and flowers (landscaping/gardening) [57]. However, results from different studies [9,27,44,45,49] indicated wider-ranging activities beyond the “4Fs” presenting a diverse population of social firms and social enterprises. As summarized in the literature, job demands may result from the design of job tasks, social relations, or the organization of work. Overall, social firms do not seem to focus on variable work environments, but rather consider social support from co-workers and supervisors as well as work organizational factors (e.g., flexible working schedules or allocation of workloads) [37].

Overall, only little research has been conducted examining specific job demands emerging in different occupational settings, for which results from the open employment market may be considered. Two examples of frequently depicted sectors include catering industry employees who described, e.g., job demands including a high work intensity, poorly predictable working hours, or overtime [58]. Cleaning workers stated an increased flexibility referring to working times (part-time work, change of work shifts at short notice) and working hours (mostly in the morning, evening, or at night). Moreover, a high work pace performing monotonous job tasks and lacking control in the work organization was highlighted [59]. Reported job demands outside social firms should be checked for transferability and analyzed for supervisors when dealing with the challenges of running a day-to-day business and tensions in balancing social and economic objectives [27,33,43,45,49,60].

### 4.2. Personal Resources and Risk Factors

In the scoping review, occupational self-efficacy was mentioned most frequently as a personal resource influencing vocational outcomes like job satisfaction, work productivity, or work engagement [36,38,39], whereas self-esteem as a worker had an impact on productivity at baseline [28]. Results outside of social firms examined the role of three personal resources (self-efficacy, organizational self-esteem, and optimism) in predicting the outcomes exhaustion and work engagement as described by Xanthopoulou et al. (2007). Personal resources were partly identified as moderating factors in the relationship between environmental resources and work engagement, suggesting that environmental resources promote the development of personal resources [61]. Encouraging social firms to continue focusing on the provision of environmental resources in addition the development of personal resources may be worthwhile when developing workplace health promotion interventions. When analyzing environmental and personal resources of employees in social firms, requirements for the diverse workforce should be considered.

Personal risk factors like a participant’s perceived severity of symptoms were displayed in the context of productivity, work engagement, and the motivation to work [28,35,38]. Further analysis of other studies on depression symptoms (besides social firms) revealed a relationship between severity of symptoms and work functioning. Even minor levels of depression were reported as having an impact on productivity [62]. Therefore, future studies should investigate the supportive work environment of social firms and the mediating effects of provided work accommodations, which may facilitate work and health-related outcomes. Since severity of symptoms showed no impact on two work engagement dimensions, participants’ willingness to work was reinforced [38] which is also in line with other studies [63]. Transferred to the already existing evidence on work engagement, it was found that engaged employees reported better health-related outcomes [64]. Torp et al. (2013) specified that work engagement mediates the relationship between control and social support on perceived depression levels. For the development of health promotion interventions, social firms should be encouraged to focus on work engagement as an important factor with a close relation to job performance [64,65].

Moreover, self-stigma was analyzed in combination with the individual work-related confidence mediating the effects of social support on work productivity [39]. Compared to results of participants in supported employment programs, reduced levels of self-stigma were reported for employees who work in non-discriminatory work environments after one year. Therefore, the relevance of a supportive work environment for target-group employees was reemphasized, in determining whether employment has positive effects on coping with self-stigma [66]. As discussed in the literature [39], psychological interventions like cognitive behavioral therapy may represent an effective approach reducing internalized negative thoughts or attitudes and improving occupational self-efficacy and therefore vocational outcomes [67].

### 4.3. Coping Strategies

Based on the concept of Lazarus and Folkman integrated into the occupational stress model of Bamberg (2003, 2006), coping strategies are considered as a buffer between stressors or work demands and health-related consequences [20]. When analyzing reported emotional and problem-focused coping strategies of the target group, it turns out that most of the applied strategies focused primary on dealing with mental illnesses of employees at work. Similar coping strategies were reported in a study conducted with participants suffering from severe mental illness in supported employment programs [68]. As can be seen from the results of Paluch et al. (2012), the complexity of the disclosure of mental illnesses affects work-related dimensions like identity, status, social interactions, and attitudes. Therefore, social firms should provide support and understanding accompanying the process of disclosure, taking social and legal consequences into account. Partnerships with support organizations, occupational health professionals, and legal experts may facilitate this responsibility [33].

Overall, results on coping strategies were mainly retrieved from qualitative studies with small sample sizes, which underlines the need for further—also quantitative—research of coping strategies of employees in social firms, when dealing with job demands [27].

### 4.4. Health-Related Outcomes

Research on the health-related outcomes of employees in social enterprises has increased over the last years. A small body of research gained insight into health-related outcomes in longitudinal studies [69,70,71,72,73,74] and for specific target groups such as homeless youth or pregnant and postpartum women enrolled in methadone treatment [70,71,72,73,74]. Additional symptom and overall improvement of health equal to the results presented in the scoping review [32,46,47] were found in a study by Jackson et al. (2009). Significantly less emergency department or ambulatory care visits as well as reduced hospital admissions were analyzed when comparing outcomes before and during training or subsequent employment in a social enterprise [69]. Resulting impacts on annual health care costs from the perspective of the public health care system were calculated comparing social firms with individuals registered in supported employment programs [75].

Moreover, two recent studies by Macaulay et al. (2018a, 2018b) examined a broader range of social enterprises with varying social missions. Strengthened social relations in the community and individual relationships, the development of social and work-related skills, confidence, and self-esteem as well as improved perceptions of health and well-being were reported [76,77]. Additional results from other comparable settings were in line [78,79,80,81,82,83,84,85,86,87], highlighting an increased employability and economic improvement [78,82,83,85,86], social participation and increasing interactive abilities [78,79,85], a sense of belonging [78,81,83,85,87], as well as high levels of job satisfaction with a positive impact on the recovery process and a possibility to disrupt adverse health behaviors [78,83,87]. Therefore, as summarized by Roy et al. (2014), social enterprises present opportunities for actions on social determinants of health referring to structural and social circumstances of individuals which impact health risks and outcomes. In the same vein, the need for evidence on causal links was claimed [88].

### 4.5. Health Promotion Interventions

Overall, health promotion research for employees in social firms is still neglected. Since job demands and resources were influenced by specific occupational settings, approaches for health promotion interventions which were transferrable to social firms need to be developed considering the needs of the diverse workforce. While a growing body of literature dealt with the provision of environmental resources in social firms, behavioral interventions should be complemented. One interventional approach for health promotion interventions was exemplified by Deforche et al. (2018) examining the effectiveness of a brief mental health promotion intervention in a social enterprise. The purpose of the intervention was to promote empowerment (main outcome), resilience, and protective factors by teaching different coping strategies (self-protection, self-care, self-acceptance, and sources of help and support) in three assignments in two group sessions. All in all, no significant effect on empowerment was observed. Significant positive effects were observed after one month on perceived social support and on palliative behavior. However, after four months follow-up adverse effects were examined on unjustified worrying, but positive effects on quality of life [89].

Other experiences were added by Hublet et al. (2016), who stated that health promotion interventions were implemented in about 65% of comparable setting like sheltered and social workshops in Belgium. Alcohol consumption (58.5%) was addressed most commonly. Actions concerning nutrition (50%), mental health (37.8%), tobacco use (36.6%), and physical activity (28%) were also of concern. Interventions were mainly designed as policy changes (43.1%), individual guidance (43.1%) or education in groups (31.5%), environmental changes (26.2%), and short running actions (11.5%). However, 55% of the health promoters reported that the implemented interventions were not adapted for people with disabilities which may impact a low-threshold attainability [90]. Therefore, literacy levels should be taken into account when developing interventions for workplace health promotion [91].

Overall, previous research indicated that a comprehensive multifactorial approach considering structural and behavioral interventions was proven to achieve most effective results [92]. A general obligation to implement workplace health promotion intervention, as recently introduced in Germany, represents a main difference compared to other companies in the open employment market.

### 4.6. Strengths and Limitations

The scoping review followed a systematic approach presented by Arksey and O’Malley (2005). To ensure a comprehensive literature search six databases were included to identify eligible studies. Afterwards, reference lists of the included studies were analyzed. Another strength of the review was the consideration of a broad spectrum of cross-sectional and longitudinal studies applying qualitative, quantitative, and mixed-method approaches. In order to facilitate the interpretation of the results of the scoping review, a quality assessment was conducted by means of the MMAT [24,25].

Nevertheless, different limitations should be addressed when transferring the results of the scoping review into policy and practice. Due to limited personnel resources, title and abstract screening was conducted by one reviewer only, whereas full texts were screened for eligibility by two reviewers independently. When interpreting the results, limited transferability resulted also from different objectives, management techniques, or legal frameworks in the contemplated countries. It should be considered that studies often referred to small sample sizes, descriptions of individual social firms, or a single type of occupational setting. In addition, the percentage of employees of a social firm with a disability ranged as well as the amount of subsidies or external funding.

Overall, the methodological quality of the included studies varied, which was therefore included in the presentation of the results. Additionally, the authors applied different measures analyzing the same construct, which may influence the comparability of the results (e.g., the assessment of the construct job satisfaction was considered using the instruments Minnesota Satisfaction Questionnaire (MSQ-SF), Warr Job satisfaction survey, or by means of single items only) [31,32,36]. Data often relied on self-report measures limiting objectivity without claiming to be representative for the target population. Most of the included studies report a cross-sectional study design (*n* = 19), which restricts the interpretation of causal links. All in all, these factors might affect the generalizability of results and the transfer to target groups in different countries.

### 4.7. Theoretical Implications

The scoping review is based on the occupational psychological stress model of Bamberg et al. (2003, 2006), which was applied in the context of the work and health situation of employees in social firms. The overview contributed to a synthesis of international research on this topic providing various starting points for further analysis and the deduction of interventions for workplace health promotion interventions.

Overall, there is a need for a comprehensive research agenda, when analyzing the work and health situation of target-group employees in social firms [93]. The inclusion of vulnerable target-groups such as people with disabilities in the research process on issues of their daily lives—as applicable to the considered occupational setting—needs to be strengthened ensuring that their perspectives are taken into account [94]. Further research activities respectively the development, implementation and evaluation of workplace health promotion interventions should be triangulated in a wider range of participants including managers, supervisors, and employees with and without disabilities.

The current body of research is characterized by a large amount of qualitative (case) studies without evidence on causal links. Therefore, more empirical research also by means of longitudinal studies will be necessary considering larger sample sizes and examining especially job demands, coping strategies or health-related outcomes, such as stress or well-being. To the latter, the included studies focus predominantly on illness reductions and rely to date mainly on cross-sectional analysis and retrospective self-assessments. Thus, conclusions about the development of clinical profiles cannot be drawn and recall biases may have influenced response behavior [32,46].

Likewise, the topic of leadership needs to be considered gaining insight into associations between different leadership styles and employee health, as well as job demands resulting from managing both economic and social processes. Future studies may also extend evidence on comparisons to other employment models such as supported employment.

Additional methodological challenges result from the heterogeneity of sectors in which social firms are located, varying company sizes, and organizational and structural characteristics [95]. Upcoming research should deal with the consideration of branch and activity specifics (such as provided products or services, hiring practices, and participatory structures [95]) in the analysis of job demands and resources of employees in this setting. To map the heterogeneity in this field and to clarify differences in structures and approaches, Lysaght et al. (2018) recently tested a tool which aims at identifying elementary dimensions of social enterprises for research and self-assessment use [96].

### 4.8. Practical Implications

The summary reflects the social and economic significance of employment in social firms for people who are able to maintain employment in a highly supportive environment. Based on the current state of research concerning working resources, job demands, coping strategies, work and health-related outcomes, and health promotion interventions, three main implications were derived.

(1) A growing body of literature suggests the provision of several environmental resources, which refer to *social support* in the first place. Several formats for facilitating the development of social support could be suggested like guidance and practical support, adapted expectations of supervisors, the support of new employees, or social events like birthday celebrations [1,27,30,34,45]. Moreover, feedback form co-workers and supervisors should be provided in formal and informal ways [40].

*Flexible working arrangements* due to scheduling and workloads should be provided including, e.g., desired working hours depending on mental and physical health, health care needs, appointments, or limits resulting from disability benefits [27,29,32,40,41,44,45,47,49]. Additionally, unscheduled breaks should be considered as well as an individual selection of shifts or an adapted work pace [29,34,40,49]. Furthermore, *meaningful work tasks* should be provided based on individual interests and abilities. Likewise, familiar routines with only a few changes in terms of work plans and locations should be considered [29,34,42,45,48].

Concepts for *training* should be developed including an adequate learning pace for new tasks or the improvement of self-management and communication skills [1]. In addition, supervisors should be provided with suitable trainings on guiding employees with mental health conditions [1].

(2) Responsible persons in social firms should focus on *employee development* as an important factor influencing future career steps [48]. Initially, an on-the-job negotiation should identify appropriate tasks and duties which fit to the employee’s skills, capacities, and interests [27,33,45]. *Monitoring* and *evaluation* activities represent elementary process steps [32,34,48], as well as *employee participation*, by means of staff meetings, workshops, committees, or related formats [42,48,50]. Currently, there are no guidelines or regulations available for the employer’s orientation [37].

(3) The last implication deals with the development of *health promotion interventions*, which are influenced by specific occupational settings and related job demands and resources. The needs of the diverse workforce should be considered (e.g., according to their literacy levels and abilities [91]) to guarantee *a low-threshold access*. Overall, a combination of structural and behavioral interventions should be implemented to improve the employees’ health effectively.

## 5. Conclusions

The results of the scoping review indicated that target-group employees of social firms often have access to several work accommodations. The included studies confirm that a mix of environmental and personal resources influenced various work-related outcomes, like work productivity, quality of work life, work engagement or job tenure, and health-related outcomes like well-being. Moreover, several job demands were reported depending on different occupational settings. Especially the areas of social relations and work organization played a major role rather than work environmental adjustments. There is a need for further research concerning the work and health situation of the diverse workforce in social firms. Additionally, more interventional studies are needed to develop and evaluate structural and behavioral interventions for workplace health promotion.

## Figures and Tables

**Table 1:** Characteristics of included studies.

	*n*	%
**Design**		
Cross-sectional	19	76.0
Longitudinal	6	24.0
**Approach**		
Qualitative	13	52.0
Quantitative	9	36.0
Mixed methods	3	12.0
**Countries**		
Canada	14	56.0
Australia	4	16.0
United Kingdom	3	12.0
Italy	3	12.0
More than one country	1	4.0
**Year of Publication**		
2000–2004	2	8.0
2005–2009	0	0.0
2010–2014	8	32.0
2015–2019	15	60.0

## Supplementary Materials

The following are available online at https://www.mdpi.com/1660-4601/17/11/3963/s1, Table S1: Example of the search strategy for the medical database PubMed, Table S2: Background information of included studies, Table S3: Quality assessment of the included studies by means of the Mixed-Methods Appraisal Tool (MMAT).

## Abbreviations

CI: confidence interval, CR: critical ratio, HR: hazard ratio, MMAT: Mixed-Methods Appraisal Tool, MSQ-SF: Minnesota Satisfaction Questionnaire, OR: odds ratio, SD: standard deviation.

## References

[1] Corbière M., Villotti P., Dewa C.S., Sultan-Taïeb H., Fraccaroli F., Zaniboni S., Durand M.-J., Lecomte T. (2019). Work accommodations in canadian social firms: Supervisors’ and workers’ perspectives. Can. J. Commun. Ment. Health.

[2] Andresen R., Oades L., Caputi P. (2003). The experience of recovery from schizophrenia: Towards an empirically validated stage model. Aust. N. Z. J. Psychiatry.

[3] Corbiere M., Lecomte T. (2009). Vocational services offered to people with severe mental illness. J. Ment. Health.

[4] Boardman J., Grove B., Perkins R., Shepherd G. (2003). Work and employment for people with psychiatric disabilities. Br. J. Psychiatry.

[5] Henry A.D., Lucca A.M. (2004). Facilitators and barriers to employment: The perspectives of people with psychiatric disabilities and employment service providers. Work.

[6] Statistik der Bundesagentur für Arbeit (2019). Situation Schwerbehinderter Menschen. Berichte: Blickpunkt Arbeitsmarkt.

[7] McQuilken M., Zahniser J.H., Novak J., Starks R.D., Olmos A., Bond G.R. (2003). The work project survey: Consumer perspectives on work. J. Vocat. Rehabil..

[8] Warner R., Mandiberg J. (2006). An update on affirmative businesses or social firms for people with mental illness. Psychiatr. Serv..

[9] Gilbert E., Marwaha S., Milton A., Johnson S., Morant N., Parsons N., Fisher A., Singh S., Cunliffe D. (2013). Social firms as a means of vocational recovery for people with mental illness: A UK survey. BMC Health Serv. Res..

[10] Bundesministerium für Arbeit und Soziales (2013). Teilhabebericht der Bundesregierung Über die Lebenslagen von Menschen mit Beeinträchtigungen, Teilhabe—Beeinträchtigung—Behinderung.

[11] Engels D., Engel H., Schmitz A. (2016). Zweiter Teilhabebericht der Bundesregierung Über die Lebenslagen von Menschen mit Beeinträchtigungen, Teilhabe—Beeinträchtigung—Behinderung.

[12] World Health Organization World Report on Disability. https://www.who.int/disabilities/world_report/2011/en/.

[13] World Health Organization Ottawa Charter for Health Promotion. http://www.euro.who.int/__data/assets/pdf_file/0004/129532/Ottawa_Charter.pdf?ua=1.

[14] Naaldenberg J., Kuijken N., van Dooren K., van Schrojenstein Lantman de Valk H. (2013). Topics, methods and challenges in health promotion for people with intellectual disabilities: A structured review of literature. Res. Dev. Disabil..

[15] Ulich E., Wülser M. (2018). Gesundheitsmanagement in Unternehmen: Arbeitspsychologische Perspektiven.

[16] Pieper C., Schröer S., Eilerts A.-L. (2019). Evidence of workplace interventions—A systematic review of systematic reviews. Int. J. Env. Res. Public Health.

[17] Bamberg E., Busch C., Ducki A., Glaveris A. (2003). Stress- und Ressourcenmanagement: Strategien und Methoden für die Neue Arbeitswelt.

[18] Bamberg E., Keller M., Wohlert C., Zeh A. (2006). BGW-Stresskonzept Das Arbeitspsychologische Stressmodell.

[19] Rohmert W., Rutenfranz J. (1975). Arbeitswissenschaftliche Beurteilung der Belastung und Beanspruchung an Unterschiedlichen Industriellen Arbeitsplätzen.

[20] Lazarus R.S., Folkman S. (1984). Stress, Appraisal, and Coping.

[21] Arksey H., O’Malley L. (2005). Scoping studies: Towards a methodological framework. Int. J. Soc. Res. Methodol..

[22] Levac D., Colquhoun H., O’Brien K.K. (2010). Scoping studies: Advancing the methodology. Implement. Sci..

[23] Tricco A.C., Lillie E., Zarin W., O’Brien K.K., Colquhoun H., Levac D., Moher D., Peters M.D.J., Horsley T., Weeks L. (2018). PRISMA Extension for scoping reviews (PRISMA-ScR): Checklist and explanation. Ann. Intern. Med..

[24] Brien S.E., Lorenzetti D.L., Lewis S., Kennedy J., Ghali W.A. (2010). Overview of a formal scoping review on health system report cards. Implement. Sci..

[25] Hong Q., Pluye P., Fàbregues S., Bartlett G., Boardman F., Cargo M., Dagenais P., Gagnon M.-P., Griffiths F., Nicolau B. (2018). Mixed Methods Appraisal Tool (MMAT), version 2018.

[26] Landis J.R., Koch G.G. (1977). The measurement of observer agreement for categorical data. Biometrics.

[27] Buhariwala P., Wilton R., Evans J. (2015). Social enterprises as enabling workplaces for people with psychiatric disabilities. Disabil. Soc..

[28] Corbière M., Zaniboni S., Dewa C.S., Villotti P., Lecomte T., Sultan-Taïeb H., Hupé J., Fraccaroli F. (2019). Work productivity of people with a psychiatric disability working in social firms. Work.

[29] Krupa T., Lagarde M., Carmichael K. (2003). Transforming sheltered workshops into affirmative businesses: An outcome evaluation. Psychiatr. Rehabil. J..

[30] Lanctôt N., Durand M.-J., Corbiere M. (2012). The quality of work life of people with severe mental disorders working in social enterprises: A qualitative study. Qual. Life Res..

[31] Lanctôt N., Corbiere M., Durand M.-J. (2012). Job tenure and quality of work life of people with psychiatric disabilities working in social enterprises. J. Vocat. Rehabil..

[32] Milton A., Parsons N., Morant N., Gilbert E., Johnson S., Fisher A., Singh S., Cunliffe D., Marwaha S. (2015). The clinical profile of employees with mental health problems working in social firms in the UK. J. Ment. Health.

[33] Paluch T., Fossey E., Harvey C. (2012). Social firms: Building cross-sectoral partnerships to create employment opportunity and supportive workplaces for people with mental illness. Work.

[34] Svanberg J., Gumley A., Wilson A. (2010). How do social firms contribute to recovery from mental illness? A qualitative study. Clin. Psychol. Psychother..

[35] Villotti P., Zaniboni S., Corbiere M., Guay S., Fraccaroli F. (2018). Reducing perceived stigma: Work integration of people with severe mental disorders in Italian social enterprise. Psychiatr. Rehabil. J..

[36] Villotti P., Corbiere M., Zaniboni S., Fraccaroli F. (2012). Individual and environmental factors related to job satisfaction in people with severe mental illness employed in social enterprises. Work.

[37] Villotti P., Corbiere M., Fossey E., Fraccaroli F., Lecomte T., Harvey C. (2017). Work accommodations and natural supports for employees with severe mental illness in social businesses: An international comparison. Community Ment. Health J..

[38] Villotti P., Balducci C., Zaniboni S., Corbiere M., Fraccaroli F. (2014). An analysis of work engagement among workers with mental disorders recently integrated to work. J. Career Assess..

[39] Villotti P., Corbiere M., Dewa C.S., Fraccaroli F., Sultan-Taieb H., Zaniboni S., Lecomte T. (2018). A serial mediation model of workplace social support on work productivity: The role of self-stigma and job tenure self-efficacy in people with severe mental disorders. Disabil. Rehabil..

[40] Williams A., Fossey E., Harvey C. (2010). Sustaining employment in a social firm: Use of the work environment impact scale v2.0 to explore views of employees with psychiatric disabilities. Br. J. Occup..

[41] Williams A., Fossey E., Harvey C. (2012). Social firms: Sustainable employment for people with mental illness. Work.

[42] Krupa T., Lysaght R. (2016). Perspectives on how social business can engender work identity among people with mental illness. J. Policy Pr..

[43] Krupa T., Sabetti J., Lysaght R. (2019). How work integration social enterprises impact the stigma of mental illness: Negotiating perceptions of legitimacy, value and competence. Soc. Enterp. J..

[44] Wilton R., Evans J. (2016). Social enterprises as spaces of encounter for mental health consumers. Area.

[45] Evans J., Wilton R. (2019). Well enough to work? Social enterprise employment and the geographies of mental health recovery. Ann. Am. Assoc. Geogr..

[46] Dewa C.S., Hoch J.S., Corbiere M., Villotti P., Trojanowski L., Sultan-Taieb H., Zaniboni S., Fraccaroli F. (2019). A Comparison of healthcare use and costs for workers with psychiatric disabilities employed in social enterprises versus those who are not employed and seeking work. Community Ment. Health J..

[47] Elmes A.I. (2019). Health impacts of a WISE: A longitudinal study. Soc. Enterp. J..

[48] Secker J., Dass S., Grove B.O.B. (2003). Developing social firms in the UK: A contribution to identifying good practice. Disabil. Soc..

[49] Wilton R., Evans J. (2018). Accounting for context: Social enterprises and meaningful employment for people with mental illness. Work.

[50] Lysaght R., Krupa T., Bouchard M. (2018). The role of social enterprise in creating work options for people with intellectual and developmental disabilities. J. Dev. Disabil..

[51] Suijkerbuijk Y.B., Schaafsma F.G., van Mechelen J.C., Ojajärvi A., Corbière M., Anema J.R. (2017). Interventions for obtaining and maintaining employment in adults with severe mental illness, a network meta-analysis. Cochrane Database Syst. Rev..

[52] Campbell K., Bond G.R., Drake R.E. (2011). Who benefits from supported employment: A meta-analytic study. Schizophr. Bull..

[53] Zaniboni S., Fraccaroli F., Villotti P., Corbiere M. (2011). Working plans of people with mental disorders employed in Italian social enterprises. Psychiatr. Rehabil. J..

[54] Chan A. (2015). Social support for improved work integration: Perspectives from Canadian social purpose enterprises. Soc. Enterp. J..

[55] Chan A. (2016). Personal wellbeing of participants of social purpose enterprises: The influence of social support. Voluntas.

[56] Gregersen S., Kuhnert S., Zimber A., Nienhaus A. (2010). Führungsverhalten und Gesundheit—Zum Stand der Forschung. Gesundheitswesen.

[57] Kirsh B., Krupa T., Cockburn L., Gewurtz R. (2006). Work initiatives for persons with severe mental illnesses in canada: A decade of development. Can. J. Commun. Ment. Health.

[58] Krüger F., Guhlemann K., Beerheide E., Georg A., Goedicke A., Nordbrock C., Seiler K. (2018). Arbeit und Arbeitsbedingungen im Gastgewerbe. Gesundheitsgerechte Dienstleistungsarbeit: Diskontinuierliche Erwerbsverläufe als Herausforderung für Arbeitsgestaltung und Kompetenzentwicklung im Gastgewerbe.

[59] EU-OSHA—European Agency for Safety and Health at Work (2009). The Occupational Safety and Health of Cleaning Workers.

[60] Costanzo L.A., Vurro C., Foster D., Servato F., Perrini F. (2014). Dual-mission management in social entrepreneurship: Qualitative evidence from social firms in the United Kingdom. J. Small Bus. Manag..

[61] Xanthopoulou D., Bakker A.B., Demerouti E., Schaufeli W.B. (2007). The role of personal resources in the job demands-resources model. Int. J. Stress Manag..

[62] Beck A., Crain L.A., Solberg L.I., Unützer J., Maciosek M.V., Whitebird R.R., Rossom R.C. (2014). Does severity of depression predict magnitude of productivity loss?. Am. J. Manag. Care.

[63] Secker B.G.P.S.J. (2001). Challenging barriers to employment, training and education for mental health service users: The service user’s perspective. J. Ment. Health.

[64] Bakker A.B. (2011). An Evidence-based model of work engagement. Curr Dir. Psychol. Sci..

[65] Torp S., Grimsmo A., Hagen S., Duran A., Gudbergsson S.B. (2013). Work engagement: A practical measure for workplace health promotion?. Health Promot. Int..

[66] Rüsch N., Nordt C., Kawohl W., Brantschen E., Bartsch B., Muller M., Corrigan P.W., Rossler W., Brantschen E.,  Bartsch B. (2014). Work-related discrimination and change in self-stigma among people with mental illness during supported employment. Psychiatr. Serv..

[67] Kukla M., Strasburger M.A., Lysaker P.H. (2016). A CBT intervention targeting competitive work outcomes for persons with mental illness. Psychiatr. Serv..

[68] Alverson M., Becker D.R., Drake R.E. (1995). An ethnographic study of coping strategies used by people with severe mental illness participating in supported employment. Psychiatr. Rehabil. J..

[69] Jackson Y., Kelland J., Cosco T.D., McNeil D.C., Reddon J.R. (2009). Nonvocational outcomes of vocational rehabilitation: Reduction in health services utilization. Work.

[70] Aklin W.M., Wong C.J., Hampton J., Svikis D.S., Stitzer M.L., Bigelow G.E., Silverman K. (2014). A therapeutic workplace for the long-term treatment of drug addiction and unemployment: Eight-year outcomes of a social business intervention. J. Subst. Abus. Treat..

[71] Ferguson K.M. (2013). Using the social enterprise intervention (SEI) and individual placement and support (IPS) models to improve employment and clinical outcomes of homeless youth with mental illness. Soc. Work Ment. Health.

[72] Ferguson K. (2012). Merging the fields of mental health and social enterprise: Lessons from abroad and cumulative findings from research with homeless youths. Community Ment. Health J..

[73] Ferguson K.M., Islam N. (2008). Conceptualizing outcomes with street-living young adults: Grounded theory approach to evaluating the social enterprise intervention. Qual. Soc. Work.

[74] Ferguson K.M. (2018). Employment outcomes from a randomized controlled trial of two employment interventions with homeless youth. J. Soc. Soc. Work Res..

[75] Sultan-Taïeb H., Villotti P., Berbiche D., Dewa C.S., Desjardins É., Fraccaroli F., Zaniboni S., Mazaniello-Chézol M., Lecomte T., Durand M.-J. (2019). Can social firms contribute to alleviating the economic burden of psychiatric disabilities for the public healthcare system?. Health Soc. Care Community.

[76] Macaulay B., Roy M.J., Donaldson C., Teasdale S., Kay A. (2018). Conceptualizing the health and well-being impacts of social enterprise: A UK-based study. Health Promot. Int..

[77] Macaulay B., Mazzei M., Roy M.J., Teasdale S., Donaldson C. (2018). Differentiating the effect of social enterprise activities on health. Soc. Sci. Med..

[78] Ho A.P.Y., Chan K.T. (2010). The social impact of work-integration social enterprise in Hong Kong. Int. Soc. Work.

[79] Lysaght R., Jakobsen K., Granhaug B. (2012). Social firms: A means for building employment skills and community integration. Work.

[80] Blonk L., Huijben T., Bredewold F., Tonkens E. (2019). Balancing care and work: A case study of recognition in a social enterprise. Disabil. Soc..

[81] Kelly D., Steiner A., Mazzei M., Baker R. (2019). Filling a void? The role of social enterprise in addressing social isolation and loneliness in rural communities. J. Rural Stud..

[82] Chan A., Ryan S., Quarter J. (2017). Supported social enterprise:A modified social welfare organization. Nonprofit Volunt. Sect. Q..

[83] Roy M.J., Baker R., Kerr S. (2017). Conceptualising the public health role of actors operating outside of formal health systems: The case of social enterprise. Soc. Sci Med..

[84] Bertotti M., Harden A., Renton A., Sheridan K. (2012). The contribution of a social enterprise to the building of social capital in a disadvantaged urban area of London. Community Dev. J..

[85] Chui C.H.-K., Shum M.H.Y., Lum T.Y.S. (2019). Work integration social enterprises as vessels of empowerment? Perspectives from employees. Asia Pac. J. Soc. Work Dev..

[86] Cho S., Kim M.A., Kwon S.I. (2019). Using the photovoice method to understand experiences of people with physical disabilities working in social enterprises. Disabil. Health J..

[87] Munoz S.A., Farmer J., Winterton R., Barraket J. (2015). The social enterprise as a space of well-being: An exploratory case study. Soc. Enterp. J..

[88] Roy M.J., Donaldson C., Baker R., Kerr S. (2014). The potential of social enterprise to enhance health and well-being: A model and systematic review. Soc. Sci. Med..

[89] Deforche B., Mommen J., Hublet A., De Roover W., Huys N., Clays E., Maes L., De Bourdeaudhuij I., Van Cauwenberg J. (2018). Evaluation of a brief intervention for promoting mental health among employees in social enterprises: A cluster randomized controlled trial. Int J. Env. Res. Public Health.

[90] Hublet A., Maes L., Mommen J., Deforche B., De Bourdeaudhuij I. (2016). Health promotion interventions in social economy companies in Flanders (Belgium). BMC Public Health.

[91] Inauen A., Jenny G.J., Bauer G.F. (2011). Design principles for data- and change-oriented organisational analysis in workplace health promotion. Health Promot. Int..

[92] Goldgruber J., Ahrens D. (2010). Effectiveness of workplace health promotion and primary prevention interventions: A review. J. Public Health.

[93] Banas J.R., Magasi S., The K., Victorson D.E. (2019). Recruiting and retaining people with disabilities for qualitative health research: Challenges and solutions. Qual. Health Res..

[94] National Disability Authority Guidelines for Including People with Disabilities in Research. http://nda.ie/nda-files/Guidelines-for-Including-People-with-Disabilities-in-Research.pdf.

[95] Roy M.J., Lysaght R., Krupa T.M. (2017). Action on the social determinants of health through social enterprise. CMAJ.

[96] Lysaght R., Roy M.J., Rendall J.S., Krupa T., Ball L., Davis J. (2018). Unpacking the foundational dimensions of work integration social enterprise the development of an assessment tool. Soc. Enterp. J..

