# Supplementary material for: Working Conditions in Social Firms and Health Promotion Interventions in Relation to Employees’ Health and Work-Related Outcomes—A Scoping Review
Source: Int J Environ Res Public Health. 2020 Jun 3;17(11):3963. doi: 10.3390/ijerph17113963 (PMC7312385; doi:10.3390/ijerph17113963)
Supplement: Supplementary file 1 [file ijerph-17-03963-s001.pdf]

Table S1. Example of the search strategy for the medical database PubMed (afterwards adapted for other databases).

**PubMed**	
#1	Social firm* [ALL] OR Consumer-run* [ALL] OR Social cooperative* [ALL] OR Social enterprise* [ALL] OR Social business* [ALL] OR Adapted enterprise* [ALL] OR Affirmative business* [ALL] OR Work integration social enterprise* [ALL]
AND	
#2	"Task characteristic" [ALL] OR "Task Performance and Analysis" [MeSH] OR Job demand* [ALL] OR Emotional demand* [ALL] OR Mental demand* [ALL] OR Physical demand* [ALL] OR "Workload" [MeSH] OR Working condition* [ALL] OR Job resource* [ALL] OR Personal resource* [ALL] OR "Resilience, psychological" [MeSH] OR "resilience" [ALL] OR Coping* [ALL] OR Strain* [ALL] OR "Stress, Psychological" [MeSH] OR Stress* [ALL] OR "Mental health" [MeSH] OR "Well-being" [ALL] OR "Occupational stress" [MeSH] OR "Occupational health" [MeSH] OR negative feeling* [ALL] OR Positive feeling* [ALL] OR "exhaustion" [ALL] OR "need for recovery" [ALL] OR "sick leave" [ALL] OR "Frustration" [MeSH] OR "sadness" [ALL] OR "negative emotions" [ALL] OR stress reaction* [ALL] OR "Burnout, Professional" [MeSH] OR burnout [ALL] OR "Depression" [MeSH] OR "Depressive Disorder" [MeSH] OR "Anxiety" [MeSH] OR "Turnover" [ALL] OR "Fatigue" [MeSH] OR "Social support" [MeSH] OR "Isolation" [ALL] OR Monoton* [ALL] OR "Work Engagement" [MeSH] OR "Work Performance" [MeSH] OR "Productivity" [ALL] OR "Job satisfaction" [MeSH] OR "Quality of work life" [ALL] OR "Motivation" [MeSH] OR "Health promotion" [MeSH] OR "Health Education" [MeSH] OR "Health offers" [ALL] OR "Stress prevention" [ALL] OR "Program Evaluation" [MeSH] OR "Program Development" [MeSH] OR "Occupational health services" [MeSH] OR "Primary Prevention" [MeSH] OR "Empowerment" [ALL] OR "Stress management" [ALL] OR "resilience training" [ALL]

Table S2. Background information of included studies.

Author, Year of Publication, Country, Reference	Study Design; Approach; Method	Setting	Sample Size (n)	Population	Main Measurements	Aim of the Study	Main Results
Buhariwala et al. (2015), Canada [27]	Cross-sectional; Qualitative; Semi-structured interviews	Social enterprises	21	Key informants (executive directors and/or managers)	Organisational structure, governance, decision-making processes, workplace accommodations, budgets and revenues	• to illustrate the range of social enterprises providing employment possibilities for individuals with psychiatric disabilities<br>• to highlight work accommodations and conditions<br>• to describe the challenges in maintaining work opportunities	• to create enabling workplaces the topics flexibility, security, support on the job, support beyond work and finding a balance between economic and social objectives were highlighted
Corbière et al. (2019b), Canada [28]	Longitudinal (follow-up after 6 months); Quantitative; Questionnaire	Social firms	222 (n = 80 from Ontario and n = 142 from Quebec)	Workers with a psychiatric disability	Severity of symptoms (Brief symptom Inventory, BSI) Self-esteem as a worker, (Rosenberg Self-Esteem as a Worker Scale, RSEWS) Organizational constraints	• to examine a theoretical model gaining insight into work productivity in the long-run taking individual and workplace-related variables into account	• work productivity was negatively influenced by severity of symptoms and organizational constraints and positively by self-esteem as a worker at baseline<br>• after six months supervisor support

Author, Year of Publication, Country, Reference	Study Design; Approach; Method	Setting	Sample Size (*n*)	Population	Main Measurements	Aim of the Study	Main Results
					(Organizational Constraints Scale, OCS) Supervisor support (Job Content Questionnaire, JCQ, supervisor support dimension) Work productivity (Endicott Productivity Scale, EWPS)		was found to be related to work productivity
Corbière et al. (2019a), Canada [1]	Cross-sectional; Quantitative; Questionnaire	Social firms (adapted enterprises and consumer/survivor-run business)	Québec: 34 supervisors, and 111 workers Ontario: 13 supervisors and 80 workers	Supervisors and workers with a mental health condition	Data on work accommodations (availability and perceived usefulness using the work accommodation and natural support scale, WANSS)	• to describe work accommodations in two types of social firms in two Canadian provinces and the perceived usefulness	• work accommodations like support from different stakeholders, the presence of a job coach, supervisor and co-worker support, training opportunities or flexible scheduling were available and perceived as useful in both types of social firms

Author, Year of Publication, Country, Reference	Study Design; Approach; Method	Setting	Sample Size (*n*)	Population	Main Measurements	Aim of the Study	Main Results
Dewa et al. (2019), Canada [46]	Cross-sectional; Quantitative; Questionnaire	Social enterprises	101 participants in social enterprises; 97 participants in the non-employed group	Workers with psychiatric disabilities and those who were not employed	Health Status (BSI, healthcare service use including psychiatric inpatient stays, emergency room (ER) visits, physician visits, and community mental health (CMH) services, prescription psychotropic medications, Client Service Receipt Inventory (CSRI) psychiatric inpatient admissions, inpatient days, and number of ER visits, physician visits (primary care and psychiatrist) and CMH program service contacts), Matryoshka	• to analyse healthcare service use and costs for social enterprise employees compared to a population of unemployed people with psychiatric disorders	• a significant difference in obtained healthcare services between the two groups were observed (during the last 6-months)<br>• employees of the social enterprise group were significantly less likely being hospitalized for a psychiatric reason or retrieving community mental health support visits

Author, Year of Publication, Country, Reference	Study Design; Approach; Method	Setting	Sample Size (*n*)	Population	Main Measurements	Aim of the Study	Main Results
					medication log, Unit Costs		
Elmes et al. (2019), Australia [47]	Longitudinal; Mixed-methods; Semi-structured interviews and quantitative questionnaire	Work integration social enterprise	31 (23 target staff and 8 managers and other staff)	Target staff and managers/other staff	Interviews: Changes in participants health, well-being, economic participation and social inclusion Questionnaire: Personal Wellbeing Index – Adult (PWI-A), RAND Short Form Health Survey (SF-36) 1.0, Kessler-10 (K-10), additional detailed custom questionnaire	• to examine the effects of a social enterprise on health outcomes	• the majority of staff reported improved health and increased well-being by means of an improving economic situation, enhanced levels of perceived confidence and social connectedness
Evans and Wilton (2019), Canada [45]	Cross-sectional; Qualitative; Semi-structured interviews	Social enterprises	88 (42 employees with mental illnesses and 46 key informants)	Key informants (executive directors and/or managers) and employees with mental illnesses	Organisational aspects and relationships between mental (ill) health and being employed in a social enterprise	• to illustrate the importance of paid work, provided work accommodations and sources of support in social enterprises	• employment experiences in social enterprises were highlighted including a meaningful activity, flexible working arrangements, job security or social support

Author, Year of Publication, Country, Reference	Study Design; Approach; Method	Setting	Sample Size (*n*)	Population	Main Measurements	Aim of the Study	Main Results
Krupa et al. (2003), Canada [29]	Cross-sectional; Mixed-methods; Focus group, questionnaire and document analysis	Affirmative business	8 focus groups with Associates (*n* = 32), one focus group with support staff (*n* = 4), survey (*n* = 73)	Affirmative businesses for people receiving mental health services	Employment outcomes of the Voices, Opportunities and Choices Employment Club (VOCEC) compared to the traditional sheltered workshop, productivity outcomes for former participants of the sheltered workshops, who did not pursue employment in VOCEC and experiences of the involvement in VOCEC	• to highlight the outcomes associated with VOCEC, which transformed to an affirmative business for people with mental health conditions (from a former sheltered workshop)	• several rewards of participation like legitimacy, ownership, illness reduction, respect and economic well-being and points of tension (financial aspects, the disability label and evolving associate control) were displayed
Krupa & Lysaght (2016), Canada [42]	Cross-sectional; Qualitative; case study methodology: Interviews, organizational documents, observation and focus groups	Social enterprises	Six businesses	Businesses created for people with mental illnesses	Business and payment structures, social value of produced goods and services, involvement of employees,	• to highlight processes and structures of social businesses and the potential to build a work identity when following social	• six business processes fostering the work identity were presented as well as propositions which can be applied for the development of social businesses

Author, Year of Publication, Country, Reference	Study Design; Approach; Method	Setting	Sample Size (*n*)	Population	Main Measurements	Aim of the Study	Main Results
					contact with the public	and economic goals	enhancing the work identity of employees
Krupa et al. (2019), Canada [43]	Cross-sectional; Qualitative; Case study methodology: Interviews, organizational documents, observation and focus groups	Work integration social enterprises	Five businesses	Businesses created for people with mental illnesses	Three social processes (perception of legitimacy, value and competence)	• to gain insight into dimensions influencing stigma associated with mental illnesses and how a social enterprise can impact those mechanisms	• different processes and tensions related to perceived legitimacy, value and competence were presented when balancing economic and social demands in a social enterprise
Lanctôt et al. (2012a), Canada [31]	Longitudinal (follow-up after 6 months); Quantitative; Questionnaire	Social enterprises	67	Workers with psychiatric disabilities	Quality of work life (Quality of work life questionnaire, QWLQ) Self-esteem as a worker (RSEWS) Job satisfaction (Minnesota Satisfaction Questionnaire Short Form, MSQ-SF) Severity of symptoms (BSI) General quality of life (Quality of Life Inventory	• to analyse the role of quality of work life on being able to hold employment for people with psychiatric disabilities employed in social enterprises	• 89% of participants were able to hold employment during the follow-up time<br>• employees, who reported a higher quality of work life had a decreased risk to terminate the employment relationship

Author, Year of Publication, Country, Reference	Study Design; Approach; Method	Setting	Sample Size (*n*)	Population	Main Measurements	Aim of the Study	Main Results
					Brief Version, QLI-BV)		
Lanctôt et al. (2012b), Canada [30]	Cross-sectional; Qualitative (phenomeno-logical study); Semi-structured interviews	Social enterprises	14	Workers with a psychiatric disability	Interpersonal and intrapersonal aspects of quality of work life and structural and physical factors	• to illustrate the views of quality of work life of people with severe mental illness working in social enterprises	• identified aspects of quality of work life included inter- and intrapersonal factors like social support from co-workers and supervisors as well as structural and physical dimensions referring e.g. to the working conditions in social enterprises
Lysaght et al. (2018), Canada [50]	Cross-sectional; Qualitative; Case study methodology: Interviews, organizational documents, observation and focus groups	Social enterprises	5 social enterprises	Businesses created for people with intellectual and developmental disabilities	Structural models, worker support models, worker engagement, social inclusion and career development	• to identify successful practices of social enterprises for employees with intellectual and developmental disabilities to enhance social inclusion	• Social enterprises offer meaningful employment and options for job preparation advancing social inclusion<br>• different challenges faced by social enterprises were presented as well as different support approaches
Milton et al. (2015),	Cross-sectional; Mixed-methods; Quantitative	Social firms	Quantitative questionnaire ($n = 80$)	Employees with mental health problems	Quantitative survey: Current and previous	• to gain insight into the clinical profile, job	• participants described low symptom and

Author, Year of Publication, Country, Reference	Study Design; Approach; Method	Setting	Sample Size (*n*)	Population	Main Measurements	Aim of the Study	Main Results
England, Wales [32]	questionnaire and semi-structured qualitative interviews (with a sub-sample)		qualitative interviews (*n* = 22)		clinical characteristics (self-stated diagnosis and types of mental health problems in the past, Brief Psychiatric Rating Scale (BPRS), Global Assessment of Functioning (GAF), Functional impairment (Disability Assessment Schedule, WHO-DAS-II), Contact with mental health services Quality of life (Manchester short assessment of quality of life, MANSA), Job satisfaction (Warr Job satisfaction survey) Qualitative Interviews:	satisfaction and views of employees with mental health problems working in social firms	disability levels, elevated levels of quality of life and job satisfaction<br>• reductions in obtained secondary mental health services were reported<br>• results concerning flexibility, the support of supervisors and colleagues and the provision of workplace accommodations, openness and acceptance, stigma, recovery and job satisfaction were displayed

Author, Year of Publication, Country, Reference	Study Design; Approach; Method	Setting	Sample Size (*n*)	Population	Main Measurements	Aim of the Study	Main Results
					nature of social firm, extent of mental health problems, length of employment, type of job and position in the business		
Paluch, et al. (2012), Australia [33]	Cross-sectional; Ethnographic study; Participant observation, interviews and document analysis	Social firm	9	Employees with and without a psychiatric disability	Experiences and understandings of the socio-cultural context in social firms	• to analyse the views of employees of a social firm in Australia	• social and business processes were described as interconnected (competition, industry pressures, droughts vs. resources, staff or time)<br>• a discourse concerning the disclosure of a mental illness was presented<br>• social supports, options for training and support as well as participatory structures were highlighted as crucial to provide supportive working conditions

Author, Year of Publication, Country, Reference	Study Design; Approach; Method	Setting	Sample Size (*n*)	Population	Main Measurements	Aim of the Study	Main Results
Secker et al. (2003), UK [48]	Cross-sectional; Qualitative; (Telephone) Interviews and focus groups	Social firms	29 managers of social firms, 13 focus group participants (Service users with an interest in work), 9 representatives of relevant agencies and 10 carers	Managers of social firms, representatives of agencies with a role in the development of the local social economy, carers of disabled people and disabled people themselves	History of social firms, wages, employee participation, socio-economic development, agencies and networks, service users and carers perspective	• to present structural and organisational factors in social firms; and to analyse the opinions of a sample of disabled service users who would like to work and those of the carers	• indicators of good practice were presented including e.g. employee participation in the development of a social firm and when running a business
Svanberg et al. (2010), Scotland [34]	Cross-sectional; Qualitative; Semi-structured interviews	Emerging social firms	16	Employees of two emerging social firms who had experienced mental illness	Recovery of mental illness and related factors in social firms	• to gain insight into employees experiences when recovering from mental illness in two emerging social firms in Scotland	• several factors were found to promote recovery like structure and flexibility without pressure, a meaningful activity with suitable challenges, leadership, group acceptance and a feeling of belonging to the social firm
Villotti et al. (2012), Italy [36]	Cross-sectional; Quantitative; Questionnaire	Social enterprises	248	Employees with severe mental disorders	Job satisfaction (single item, Psychiatric Rehabilitation Determination Instrument),	• to gain insight into the influence of individual and environmental factors on job satisfaction of	• workers with higher levels of occupational self-efficacy who had access to work accommodations

Author, Year of Publication, Country, Reference	Study Design; Approach; Method	Setting	Sample Size (*n*)	Population	Main Measurements	Aim of the Study	Main Results
					Workplace accommodations (Work accommodation Inventory), Social support dimensions (JCQ), Perceived Stress Scale, PSS), Organizational constraints (OCS), Severity of symptoms (BSI), Occupational self-efficacy (Occupational self-efficacy scale)	employees with severe mental illness in social enterprises	and were provided with social support were more likely to express higher job satisfaction
Villotti et al. (2014), Italy [38]	Longitudinal (follow-up after 12 months); Quantitative; Questionnaire	Social enterprises	121	Employees with severe mental disorders	Work engagement (Utrecht Work Engagement scale, UWES-9), Severity of symptoms (BSI), Social support (JCQ), Occupational self-efficacy (Occupational	• to examine the validity of the work engagement construct and to gain insight into the relationship of work engagement with its predictors and its consequences	• employees working in social enterprises who were provided with support from colleagues and supervisors were dedicated and absorbed in their work-related tasks<br>• occupational self-efficacy was linked to the three

Author, Year of Publication, Country, Reference	Study Design; Approach; Method	Setting	Sample Size (*n*)	Population	Main Measurements	Aim of the Study	Main Results
					self-efficacy short), Future working plans (two items to examine the intention to work in private or public sector of the regular labour market)	for workers with a mental illness	dimensions of work engagement<br>• work engagement (dimension vigour) influenced the plan to work in the open labour market
Villotti et al. (2017), Italy, Australia, Canada [37]	Cross-sectional; Quantitative; Questionnaire	Social businesses	90 (Australia (*n* = 30), Canada (*n* = 30) and Italy (*n* = 30)	Employees with self-reported psychiatric disabilities	Work accommodations and natural supports (WANSS)	• to analyse provided work accommodations and natural supports in social businesses in Australia, Canada and Italy	• in all countries considered, many work accommodations and natural supports like flexibility, opportunities for training, support and feedback form supervisors and co-workers were provided<br>• accommodations like training and schedule flexibility were related to an extended job tenure
Villotti et al. (2018a), Canada [39]	Longitudinal (follow-up after 6 months);	Social enterprises	170	Employees with severe mental disorders	Social support (JCQ), Self-stigma (Internalized	• to examine the relationship between social support and	• an indirect relationship between high levels of workplace social

Author, Year of Publication, Country, Reference	Study Design; Approach; Method	Setting	Sample Size (*n*)	Population	Main Measurements	Aim of the Study	Main Results
	Quantitative; Questionnaire				Stigma of Mental Illness scale), Job tenure self-efficacy (Job tenure Self-Efficacy scale), Work productivity (EWPS)	productivity considering the mediators self-stigma and job tenure self-efficacy of employees with severe mental illness in social enterprises	support and self-perceived productivity was found to be mediated by self-stigma and confidence in facing job-related problems
Villotti et al. (2018b), Italy [35]	Longitudinal (follow-up after 12 months); Quantitative; Questionnaire	Social enterprises	139	Employees with severe mental disorders	Severity of symptoms (BSI), Occupational self-efficacy (Occupational self-efficacy short), Organizational constraints (OCS), Social support (JCQ), Motivation to Keep a Job scale, Improved work social skills (scale created and discussed by experts), Work productivity (EWPS), Perceived	• to gain insight into perceptions of being stigmatized and the reduction of discrimination by means of work experiences in social enterprises	• results demonstrated that working in a social enterprise increased working and social skills, which in turn led to elevated perceived productivity and a reduction of self-reported discrimination and stigmatization after 12 months follow up

Author, Year of Publication, Country, Reference	Study Design; Approach; Method	Setting	Sample Size (*n*)	Population	Main Measurements	Aim of the Study	Main Results
					stigma and discrimination (Stigma Scale)		
Williams et al. (2010), Australia [40]	Cross-sectional; Qualitative; Semi-structured interviews	Social firm	7	Employees who experienced persisted mental illness	Work environment impact scale (v2.0)	• to examine the views of workers with a mental health illness about the working conditions in a social firm	• working in social firms was perceived as positive, when provided with a regular structure and accomplishable tasks, social support and several rewards of the job like remuneration
Williams et al. (2012), Australia [41]	Cross-sectional; Qualitative; Semi-structured interviews	Social firm	7	Employees who experienced persisted mental illness	Work environment impact scale (v2.0), Job satisfaction (revised version of the Indiana Job Satisfaction scale, CV-IJSS)	• to identify the experiences of people with mental health conditions about their employment in a social firm and supportive working conditions	• supportive factors were presented including benefits of the job like remuneration, structures work tasks, flexible work arrangements and benevolent workplace interactions
Wilton and Evans (2016), Canada [44]	Cross-sectional; Qualitative; Semi-structured interviews and focus groups	Social enterprises	46 key informants, 63 employees, 4 focus groups involving 22 workers	key informants (executive director or manager) and employees with a mental health condition	Social enterprises as sites of encounter	• to inform about how and to what extent social enterprises create supportive environments	• different approaches to accommodate were presented as well as the provision of support during

Author, Year of Publication, Country, Reference	Study Design; Approach; Method	Setting	Sample Size (*n*)	Population	Main Measurements	Aim of the Study	Main Results
						enabling social interactions	work-related social interactions
Wilton and Evans (2018), Canada [49]	Cross-sectional; Qualitative; Semi-structured interviews	Social enterprises	46	key informants (executive director and/or manager)	Work environment and provided accommodations	• to increase an understanding of social enterprises from an organisational point of view	• the creation of meaningful employment, hours of work and remuneration were presented as well as the nature of work accommodations including flexibility, security and social support

Abbreviations: BPRS: Brief Psychiatric Rating Scale, BSI: Brief symptom Inventory, CV-IJSS: Indiana Job Satisfaction scale, EWPS: Endicott Productivity Scale, GAF: Global Assessment of Functioning, JCQ: Job Content Questionnaire, K-10: Kessler-10, MANSA: Manchester short assessment of quality of life, MSQ-SF: Minnesota Satisfaction Questionnaire Short Form, OCS: Organizational Constraints Scale, PSS: Perceived Stress Scale, PWI-A: Wellbeing Index – Adult, QLI-BV: Quality of Life Inventory Brief Version, QWLQ: Quality of work life questionnaire, RAND SF-36: Short Form Health Survey 1.0, RSEWS: Rosenberg Self-Esteem as a Worker Scale, UWES-9: Utrecht Work Engagement scale, VOCEC: Voices, Opportunities and Choices Employment Club, WANSS: Work accommodation and natural support scale, WES: Work Environment Scale, WHO-DAS-II: Disability Assessment Schedule

Table S3. Quality assessment of the included studies by means of the Mixed Methods Appraisal Tool (MMAT).

		Burhariwala et al. (2015) [27]	Corbière et al. (2019b) [28]	Corbière et al. (2019a) [1]	Dewa et al. (2019) [46]	Elmes et al. (2019) [47]	Evans & Wilton (2019) [45]	Krupa et al. (2003) [29]	Krupa et al. (2016) [42]	Krupa et al. (2019) [43]	Lanctôt et al. (2012a) [31]	Lanctôt et al. (2012b) [30]	Lysaght et al. (2018) [50]	Milton et al. (2015) [32]	Paluch et al. (2012) [33]	Secker et al. (2003) [48]	Svanberg et al. (2010) [34]	Villotti et al. (2012) [36]	Villotti et al. (2014) [38]	Villotti et al. (2018a) [39]	Villotti et al. (2018b) [35]	Villotti et al. (2017) [37]	Williams et al. (2010) [40]	Williams et al. (2012) [41]	Wilton & Evans (2016) [44]	Wilton & Evans (2018) [49]
Screening questions (for all types)	S1. Are there clear research questions?	✔	✔	✔	✔	✔	✔	✔	✔	✔	✔	✔	✔	✔	✔	✔	✔	✔	✔	✔	✔	✔	✔	✔	✔	✔
	S2. Do the collected data allow to address the research questions?	✔	✔	✔	✔	✔	✔	✔	✔	✔	✔	✔	✔	✔	✔	✔	✔	✔	✔	✔	✔	✔	✔	✔	✔	✔
Qualitative	1.1. Is the qualitative approach appropriate to answer the research question?	✔				✔	✔	✔	✔	✔		✔	✔	✔	✔	✔	✔						✔	✔	✔	✔
	1.2. Are the qualitative	✔				✔	✔	✔	✔	✔		✔	✔	✔	✔	✔	✔						✔	✔	✔	✔

data collection methods adequate to address the research question?																									
1.3. Are the findings adequately derived from the data?	✔				✔	–	✔	-	✔		✔	✔	✔	✔	–	✔						✔	✔	-	✔
1.4. Is the inter-pretation of results sufficiently sub-stantiated by data?	✔				✔	✔	✔	✔	✔		✔	✗	✗	✔	✔	✔						✔	✔	✔	✔
1.5. Is there coherence between qualitative data sources, collection, analysis and inter-pretation?	✔				✔	✗	✔	✗	✔		✔	✗	✗	✔	✗	✔						✔	✔	✗	✔

Quantitative randomized controlled trials	2.1. Is randomization appropriately performed?	
	2.2. Are the groups comparable at baseline?	
	2.3. Are there complete outcome data?	
	2.4. Are outcome assessors blinded to the intervention provided?	
	2.5 Did the participants adhere to the assigned intervention?	
Quantitative non-	3.1. Are the participants representative	✗

	of the target population?	
	3.2. Are measurements appropriate regarding both the outcome and intervention (or exposure)?	✔
	3.3. Are there complete outcome data?	✔
	3.4. Are the confounders accounted for in the design and analysis?	✔
	3.5. During the study period, is the intervention administered (or	✔

	exposure occurred) as intended?																				
Quantitative descriptive	4.1. Is the sampling strategy relevant to address the research question?	✗	✗		✔		✗			✗			✗				✗	✗	✗	✗	✗
	4.2. Is the sample re-presentative of the target population?	✗	✗		✔		–			✗			✗				✗	✗	✗	✗	✗
	4.3. Are the measure-ments appropriate?	✔	✔		✔		✔			✔			✔				✔	✔	✔	✔	✔
	4.4. Is the risk of nonrespons e bias low?	–	–		✔		–			–			✔				–	–	–	–	–
	4.5. Is the statistical analysis appropriate to answer the research question?	✔	✔		✗		✔			✔			✔				✔	✔	✔	✔	✔
Mi xe	5.1. Is there an adequate				✔		✗						✔								

rationale for using a mixed methods design to address the research question?			
5.2. Are the different components of the study effectively integrated to answer the research question?	✔	✔	✗
5.3. Are the outputs of the integration of qualitative and quantitative components adequately interpreted?	✗	✗	✗
5.4. Are divergences and incon-sistencies between	✗	✔	✔

	quantitative and qualitative results adequately addressed?					
	5.5. Do the different components of the study adhere to the quality criteria of each tradition of the methods involved?	✗	✗			✗

✓ = Yes, ✗ = no, – = Can’t tell
